# Ferroptosis in arterial atherosclerosis: mechanistic hypotheses, cell type specific vulnerabilities, translational biomarkers, and therapeutic opportunities

**DOI:** 10.3389/fimmu.2026.1868492

**Published:** 2026-06-24

**Authors:** Cai Li, Jinxia Wang, Jingyi Sun

**Affiliations:** 1 College of Basic Medical Science, Qilu Medical University, Zibo, Shandong, China; 2 Department of Clinical Laboratory, Binzhou Medical University Hospital, Binzhou, Shandong, China

**Keywords:** atherosclerosis, endothelial dysfunction, ferroptosis, iron metabolism, lipid peroxidation, oxidized phospholipids, plaque instability

## Abstract

Despite significant progress in lipid-lowering and anti-thrombotic therapies, atherosclerosis remains a leading cause of myocardial infarctions and ischemic strokes. Residual risk persists, often linked to sustained vascular inflammation, oxidative stress, and plaque instability—processes that converge on lipid peroxidation pathways within the arterial wall. Ferroptosis, an iron-dependent form of regulated cell death, occurs when antioxidant defenses fail, leading to toxic accumulation of phospholipid peroxides. Atherosclerotic plaques provide a permissive microenvironment rich in oxidized lipids, redox-active iron, inflammatory mediators, and hypoxia, suggesting ferroptosis may contribute to endothelial barrier dysfunction, macrophage foam cell death, vascular smooth muscle cell loss, and necrotic core expansion. However, current understanding is constrained by operational definition inconsistencies, reliance on non-specific oxidative stress markers, and insufficient validation in human plaques. This review systematically synthesizes knowledge on ferroptosis in arterial atherosclerosis, incorporating core pathway dynamics—iron homeostasis, polyunsaturated phospholipid metabolism, and lipid peroxide detoxification via systems like Xc–GPX4—alongside parallel protective axes. It evaluates cell-type-specific vulnerabilities across disease stages, highlighting how disturbed flow, dyslipidemia, metabolic disorders, and innate immune signaling modulate ferroptosis susceptibility and plaque phenotype. The analysis extends to candidate biomarkers, tissue-level signatures, and therapeutic strategies targeting iron availability, lipid peroxidation, or key regulatory proteins, while addressing safety concerns and experimental gaps. Ultimately, delineating ferroptosis-specific signatures in human tissues and establishing causality through cell-targeted interventions are vital for translating this pathway into clinically actionable risk assessment and management strategies.

## Introduction

### Clinical and biological context of atherosclerosis

Arterial atherosclerosis is an ongoing inflammatory process within the vessel wall; it is responsible for the majority of myocardial infarcts, ischemic strokes and peripheral arterial events (1–3). Lowering levels of apolipoprotein B-containing lipoproteins remains the foundation for prevention of the disease (4–6). However, despite successful lowering of plasma lipids, there exists a significant amount of residual risk in many patient populations, including those with diabetes mellitus, chronic kidney disease, smoking history and evidence of systemic inflammation.

Furthermore, emerging evidence reveals that the temporal patternof lipid exposure is as critical as absolute lipid levels. A pivotal 2025 study by van der Valk et al. demonstrated that early intermittent hyperlipidemia acts as a distinct atherosclerotic driver, independent of sustained hypercholesterolemia (7). This oscillatory cholesterol exposure accelerates plaque formation through IL-1β-dependent neutrophil reprogramming, which establishes a pro-inflammatory milieu that impairs macrophage homeostatic functions—specifically disrupting autophagy and efferocytosis. These findings have profound implications for ferroptosis susceptibility: IL-1β is known to prime macrophages for ferroptosis by upregulating iron uptake and suppressing antioxidant defenses. Notably, this relationship is bidirectional in atherosclerotic foam cells, where ferroptosis itself drives IL-1β production, creating a positive feedback loop that amplifies local inflammation and further lowers ferroptosis thresholds (8), While defective efferocytosis and autophagy impairment release redox-active iron, expanding the labile iron pool required for ferroptotic execution. The mechanistic link between oscillatory metabolic stress and labile iron expansion is mediated by NCOA4-dependent ferritinophagy, which degrades ferritin to release redox-active iron specifically under conditions of impaired autophagic flux (9). Furthermore, defective efferocytosis—a hallmark of intermittent hyperlipidemia—converts macrophage ferroptosis from a contained, resolvable event into a driver of progressive necrotic core expansion, as uncleared ferroptotic cells release catalytic iron and oxidized phospholipids that propagate lipid peroxidation to neighboring cells (10). We therefore hypothesize that these oscillatory metabolic conditions lower ferroptosis thresholds in plaque-resident cells by disrupting adaptive redox responses and iron handling, setting the stage for the oxidative phospholipid stress and iron-dependent cell death that characterize advanced vulnerable plaques.

The residual risk is not entirely due to the amount of lipid present at the tissue level but also due to the “quality” of the plaque, which includes the competence of the endothelium, immune cell activation, the size of the necrotic core, integrity of the fibrous cap and propensity for thrombus formation (11). Oxidative phospholipid stress has been identified as a common lesion feature among all of these mechanisms. This is evidenced through oxidation of lipoproteins (such as LDL), the presence of reactive lipid aldehydes, and the presence of redox active iron, creating an environment in which lipid radicals can initiate radical chain reactions. These findings have motivated investigators to focus on programmed cell death mechanisms that result from lipid peroxidation.

### From oxidative injury to regulated cell death programs in vascular pathology

Cell death is not simply the result of plaque mechanical damage. It is an important factor in determining the architectural structure of a lesion and its clinical behavior. In the initial stages of atherosclerotic lesions, apoptosis may be offset by highly efficient phagocytic removal (efferocytosis), while in later stages, failure of this removal leads to conversion of apoptosis into secondary necrosis and expansion of the necrotic core (12–14). Other programmed cell death mechanisms, such as pyroptosis and necroptosis, relate inflammatory signal transduction pathways to cell membrane disruption and cytokine production, and have been implicated in both the progression and instability of atherosclerotic plaques (15–17). Nevertheless, many studies employing oxidative injury models in atherosclerosis utilized non-specific endpoints, for example, overall accumulation of reactive oxygen species, or generation of malondialdehyde; these were performed with little mechanistic basis to explain how lipid oxidation will lead to a regulated and lethal cell death process. The identification of ferroptosis as a unique, genetically and pharmacologically accessible cell death pathway has provided a mechanistic link between dyslipidemia, iron chemistry, and inflammation-induced changes in atherosclerotic plaques.

### Ferroptosis: definition & plaque permissiveness

Ferroptosis is defined as an organized, iron-dependent form of regulated cell death executed by the toxic accumulation of oxidized phospholipids in cell membranes, distinct from other death pathways like apoptosis, pyroptosis, or necroptosis (18–20). The atherosclerotic plaque microenvironment is highly permissive to this process because it concurrently provides the essential components: abundant peroxidizable lipid substrates from modified lipoproteins and membrane remodeling (21–23), catalytic iron from dysregulated metabolism and intraplaque hemorrhage (24–26), and impaired antioxidant defenses due to inflammation and hypoxia which cripple systems like Xc-/GPX4. Consequently, establishing ferroptosis in vascular research requires converging evidence beyond generic oxidative stress markers, specifically: 1) demonstrating accumulation of phospholipid hydroperoxides via targeted assays, 2) documenting the essential role of iron through chelation or metabolic manipulation, and 3) showing specific rescue with ferroptosis inhibitors that block lipid radical propagation or restore detoxification, ideally combined with genetic perturbation of key regulators like GPX4. This multi-domain validation is critical due to the pleiotropic effects of many inhibitors and the overlapping oxidative phenotypes in plaques.

Beyond static plaque features, the dynamic history of metabolic exposures—specifically intermittent or oscillatory hyperlipidemia—critically shapes ferroptosis vulnerability. Cabrera-Fuentes and Boisvert demonstrated that oscillatory cholesterol exposure uniquely accelerates atherosclerosis through IL-1β-dependent neutrophil reprogramming, driving sustained macrophage dysfunction, autophagy impairment, and efferocytosis deficits (7). These processes directly intersect with ferroptosis execution pathways: repeated challenges to the Xc^-^-GSH-GPX4 axis without recovery periods may deplete antioxidant reserves (27), while impaired efferocytosis prevents clearance of ferroptotic foam cells, converting contained death into propagating inflammation. Furthermore, metabolic stress impairs NRF2-mediated antioxidant defenses and disrupts autophagic flux, creating a permissive environment for ferroptosis in atherosclerotic lesions (28). The intersection of intermittent hyperlipidemia with ferroptosis thresholds remains unexplored but represents a priority for translational research.

### Cell type specific relevance in atherosclerosis

Ferroptosis exhibits cell-type-specific impacts in the multi-cellular context of atherosclerosis. Endothelial cells at the blood-vessel interface, exposed to disturbed flow and oxidative lipids, are susceptible to ferroptosis, which can disrupt barrier function and promote pro-thrombotic signaling (29–31). Macrophages and foam cells, central to lipid uptake and inflammation, possess plastic iron metabolism; their ferroptosis can seed the necrotic core, impair dead cell clearance (efferocytosis), and amplify local inflammation through the release of oxidized lipids and catalytic iron (18–20). Vascular smooth muscle cells (VSMCs), crucial for synthesizing the collagen-rich fibrous cap, are sensitive to redox stress; their loss via ferroptosis directly weakens cap integrity, increasing plaque rupture risk (23, 32, 33).

## Core ferroptosis circuitry in the arterial wall

Ferroptosis can be thought of as an inability to manage peroxide on cell membranes when there is enough iron to catalyze and enough of the appropriate phospholipids for peroxidation in excess of what cells are able to detoxify (21). In the artery wall this balance is formed through dynamic interactions of lipoproteins bringing lipid into the wall; inflammatory redox signals indicating how much oxidative stress exists; and iron handling varies greatly among endothelial cells, macrophage foam cells and vascular smooth muscle cells.

**Figure 1:**
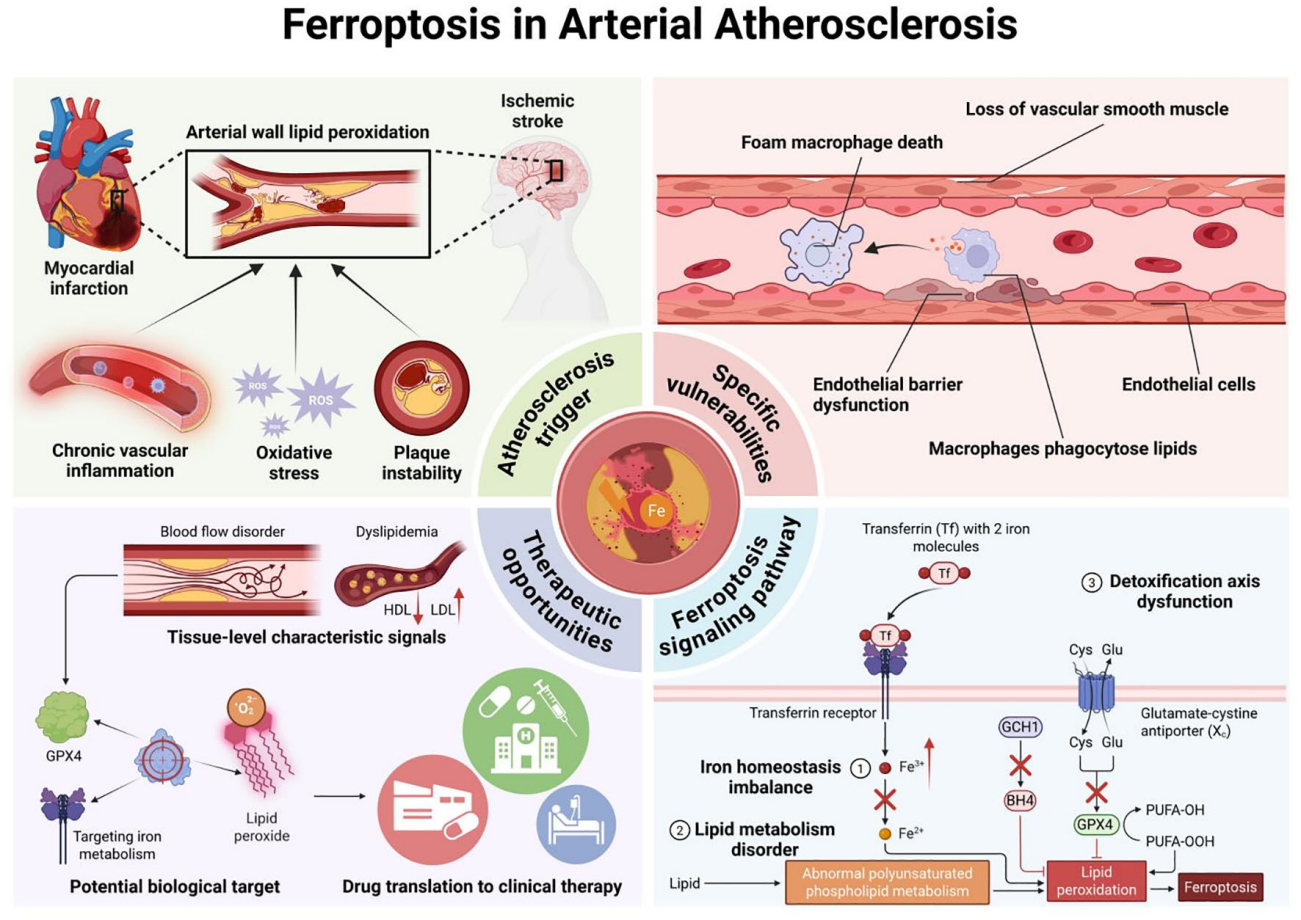
Iron overload and ox-LDL drive ferroptosis in atherosclerosis via ROS production and GPX4-dependent lipid peroxidation, promoting M1 polarization and plaque progression.

To provide a unifying mechanistic frame for later sections, **Figure 1** maps plaque microenvironment features, including oxidized lipoproteins, hypoxia, inflammatory cytokines, and iron deposition, onto the major ferroptosis checkpoints.

### Iron handling and catalytic redox permissiveness in plaques

Ferroptosis requires catalytically active iron, not total iron. In plaques, macrophages determine labile iron pool size through iron uptake (transferrin receptor), storage (ferritin), and release (ferroportin). Their location in lipid-rich lesions makes them key regulators of ferroptosis susceptibility (24–26). This degree of influence is due to their ability to control the amount of iron entering cells, the storage capacity of ferritin for iron, and the amount of iron being released back into circulation through the actions of ferroportin. Thus, the combination of these mechanisms determines whether iron is sequestered (and thus rendered non-redox active) or is in a state where it can undergo redox cycling and contribute to lipid peroxidation. Additionally, ferritinophagy can release sequestered iron and increase the labile pool of iron during times of stress and can provide a link between inflammatory signaling, autophagic remodeling and ferroptosis sensitivity.

### Phospholipid substrate availability and membrane remodeling

Ferroptosis is mediated by oxidized phospholipids, but not by lipid peroxidation. The type and quantity of peroxidizable phospholipid substrates will influence how lipid radicals will amplify and form fatal levels of membrane damage (29, 34, 35). Atherosclerotic diseases modify the amount of lipid substrates available for the formation of membrane phospholipids through modifications in uptake and recycling of lipoproteins, intracellular lipid transport and modification of membrane composition due to inflammation. Phospholipids containing polyunsaturated fatty acids have a greater propensity for oxidation because of the presence of two adjacent bis allylic hydrogen atoms which allow for the generation of rapidly propagating free radicals. Therefore, cellular enzymes involved in activating long chain fatty acids and their incorporation into phospholipids act as upstream regulators of ferroptosis susceptibility by increasing the total number of peroxidizable membrane lipids (30, 31, 36).

Lipid substrates can become oxidized through enzymatic or non-enzymatic means. Inflammation can lead to increased steady-state concentrations of lipid hydroperoxides through the oxidation of lipid substrates by lipid oxygenation pathways, whereas iron can catalyze the conversion of lipid hydroperoxides to reactive radicals capable of driving membrane bilayer chain reaction (37–39). The atherosclerotic disease process provides a unique biochemical microenvironment in which both immune activation and iron availability can occur simultaneously, leading to conditions where lipid hydroperoxide accumulation occurs at an accelerated rate and lipid radical chains may propagate unimpeded. Methods used to quantify phospholipid hydroperoxides or oxidized phosphatidylethanolamine and related species offer greater specificity than methods that utilize thiobarbituric acid reactive substances, such as malondialdehyde, as surrogate measures of oxidative stress.

### Antioxidant defense modules that suppress ferroptosis

Cells prevent ferroptosis by a multi-layered defense strategy based on removal of phospholipid hydroperoxides and interruption of radical chains. The major component of the first layer is the Xc- system, glutathione(GSH) and glutathione peroxidase 4 (GPx4) axis (18–20). The cysteine uptake via the Xc- system allows cells to synthesize GSH; GPx4 removes phospholipid hydroperoxides from membranes and prevents their transformation into radicals that amplify radical production. However, the Xc- system/GSH/GPx4 module is sensitive to nutritional deficiencies, as well as the effects of inflammation, which lead to changes in cellular metabolism and an increased load of peroxides. In addition, in later stages of atherosclerotic lesion development, factors such as reduced oxygen availability, alterations in redox status and reduced ability of the micro-environment to sustain the protective role of this module, result in a reduction in the safety margin for this pathway (33, 40, 41).

There exist parallel pathways to inhibit ferroptosis, some of which may be more active than others depending on cell-type and compartment. One of these pathways involves the enzyme ferroxidase FSP1, which traps radicals at membranes using coenzyme Q as its electron acceptor, thereby providing a GPx4-independent mechanism of inhibition (32, 42, 43). Another pathway involves the GCH1/BH4 system, which controls lipid antioxidant activity and determines the susceptibility of cells to peroxidation (42, 44, 45). Mitochondrial-linked mechanisms can also provide protection against ferroptosis, especially when there is overlap between mitochondrial redox flux and lipid peroxidation in membranes, but the extent to which mitochondria are required vs. permissible to allow ferroptosis to proceed will depend upon both the model used and the nature of the insult. Finally, transcriptional programs that control iron homeostasis, cystine uptake and the expression of detoxifying enzymes (such as those regulated by NRF2) represent a systems-level approach that links inflammatory and metabolic signals to the regulation of ferroptosis thresholds.

### Pharmacologic and genetic validation logic in plaque relevant systems

One of the main problems with the literature about atherosclerosis is that many researchers tend to overstate the role of antioxidants in modulating ferroptosis, simply because many agents that can lower oxidative stress have other mechanisms of action (e.g., anti-inflammatory, lipid metabolic changes, changes in mitochondria). In order to increase the level of mechanistic confidence for ferroptosis in vascular studies, it would be best if three criteria were met: 1) The agent used has been shown to affect one of the key regulators of ferroptosis (GPX4, SLC7A11, ACSL4, or FSP1); 2) The agent was shown to depend on iron levels through either perturbing iron handling or using an iron chelator; 3) The agent decreased the amount of phospholipid hydroperoxides present and rescued cell viability or plaque relevant phenotypes. By having at least two of these criteria met, studies will be better able to conclude that their results are due to ferroptosis modulation rather than just lowering oxidative stress. Consistent with these principles, the evidence synthesized below is explicitly evaluated against these three domains. Studies satisfying all three criteria are interpreted as providing robust support for ferroptosis involvement, whereas those fulfilling only one or two domains are regarded as suggestive but mechanistically incomplete.

To standardize how this review interprets pathway evidence across models and cell types, the key regulators, preferred readouts, and translational implications are summarized in **Table 1**.

**Table 1:** Ferroptosis regulators relevant to atherosclerosis and lesion linked implications.

Axis or regulator	Primary functional role in ferroptosis	Predicted plaque relevant effect if activated or increased	Predicted plaque relevant effect if inhibited or decreased	Preferred mechanistic readouts	Translational notes and intervention logic
Labile iron pool (46)	Catalytic driver of lipid radical formation and propagation	Increased iron permissiveness promotes lipid peroxide amplification and ferroptotic commitment	Reduced catalytic iron raises ferroptosis threshold	Labile iron probes, iron redox mapping, ferroptosis rescue with chelators	Therapeutic iron modulation must balance systemic iron needs and infection risk
Transferrin receptor pathway (22)	Iron uptake and intracellular availability	Enhanced uptake can expand labile pools in permissive microdomains	Reduced uptake lowers iron driven lipid oxidation	Uptake assays, receptor expression with labile iron confirmation	Targeting uptake may be cell type specific, especially in macrophage rich lesions
Ferritin and ferritinophagy (47)	Iron buffering versus iron mobilization	Mobilization of ferritin iron can increase labile pools under stress	Increased buffering capacity can be protective	Ferritin levels, ferritinophagy markers, labile iron changes under perturbation	Context dependent, buffering is protective, mobilization may be deleterious in lesions
Ferroportin dependent export (48)	Iron efflux and iron retention control	Reduced export increases intracellular retention and potential catalytic pools	Enhanced export decreases intracellular catalytic iron	Export assays, intracellular labile iron, effects of hepcidin like signaling	Lesion macrophage iron retention may couple inflammation to ferroptosis sensitivity
Heme handling and heme catabolism (49)	Converts heme to iron and metabolites, can be protective or permissive	Excess heme breakdown can increase iron availability in hemorrhagic niches	Controlled buffering and clearance may be protective	Heme markers, iron localization, coupling to lipid peroxidation signatures	Particularly relevant in hemorrhage prone plaques and erythrocyte rich regions
ACSL4 (50)	Enriches membranes with peroxidizable polyunsaturated phospholipids	Increased peroxidizable substrate supply sensitizes to ferroptosis	Reduced substrate enrichment reduces susceptibility	Phospholipid composition, oxidized phospholipid species, genetic perturbation	Strong mechanistic lever, but impacts broader lipid metabolism
LPCAT3 (51)	Incorporates polyunsaturated fatty acids into phospholipids	Promotes vulnerable phospholipid pools that support peroxide accumulation	Decreases peroxidizable phospholipid abundance	Lipidomics of membrane phospholipids, hydroperoxide quantification	May be cell type specific because membrane remodeling differs across plaque cells
System Xc minus, SLC7A11 (20)	Cystine import supporting glutathione synthesis	Increased activity strengthens peroxide detoxification capacity	Decreased activity depletes glutathione and sensitizes	Cystine uptake, glutathione levels, selective rescue experiments	Nutrient limitation and inflammatory metabolism can functionally suppress this pathway
Glutathione, GSH (52)	Reducing equivalent for GPX4 activity	Supports detoxification of phospholipid hydroperoxides	Depletion lowers GPX4 effectiveness	GSH and GSSG, redox state, GPX4 dependent rescue patterns	GSH depletion is not specific, must be paired with phospholipid peroxide evidence
GPX4 (53)	Direct reduction of phospholipid hydroperoxides in membranes	Increased activity blocks execution phase	Loss or inhibition is a potent trigger	GPX4 protein and activity assays, lipid hydroperoxide reduction, genetic tests	Central checkpoint, but manipulation may have systemic toxicity without targeting
FSP1 and CoQ (32)	GPX4 independent radical trapping at membranes	Strengthens membrane defense against lipid radical propagation	Weakening increases vulnerability	CoQ redox state, FSP1 perturbation, rescue logic independent of GPX4	Potential therapeutic axis when GPX4 pathways are compromised
GCH1 (54)	Antioxidant capacity shaping lipid peroxidation susceptibility	Reinforces suppression of lipid oxidation and ferroptosis	Reduction increases susceptibility	BH4 quantification, oxidized lipid markers, pathway perturbation	Links immunometabolism and redox regulation to ferroptosis thresholds
NRF2 program (55)	Coordinates iron buffering and antioxidant gene expression	Activation increases resilience to ferroptotic stress	Suppression increases vulnerability	Transcript and protein panels, functional assays under ferroptotic stress	NRF2 activation is broad, specificity requires downstream ferroptosis readouts

## Ferroptosis across atherosclerosis stages and plaque microenvironments

Atherosclerosis represents a series of different lesion states (microenvironments) as opposed to being one single lesion state; these microenvironments are characterized by differing levels of lipid load, inflammation, oxygen availability and iron availability, at different times and locations within the plaque (56). The purpose of this section is to identify areas of the plaque where ferroptosis is most likely to occur based upon characteristics of different lesion stages and microenvironmental niches, and correlate those locations to plaque types that predict clinical outcomes.

**Figure 2:**
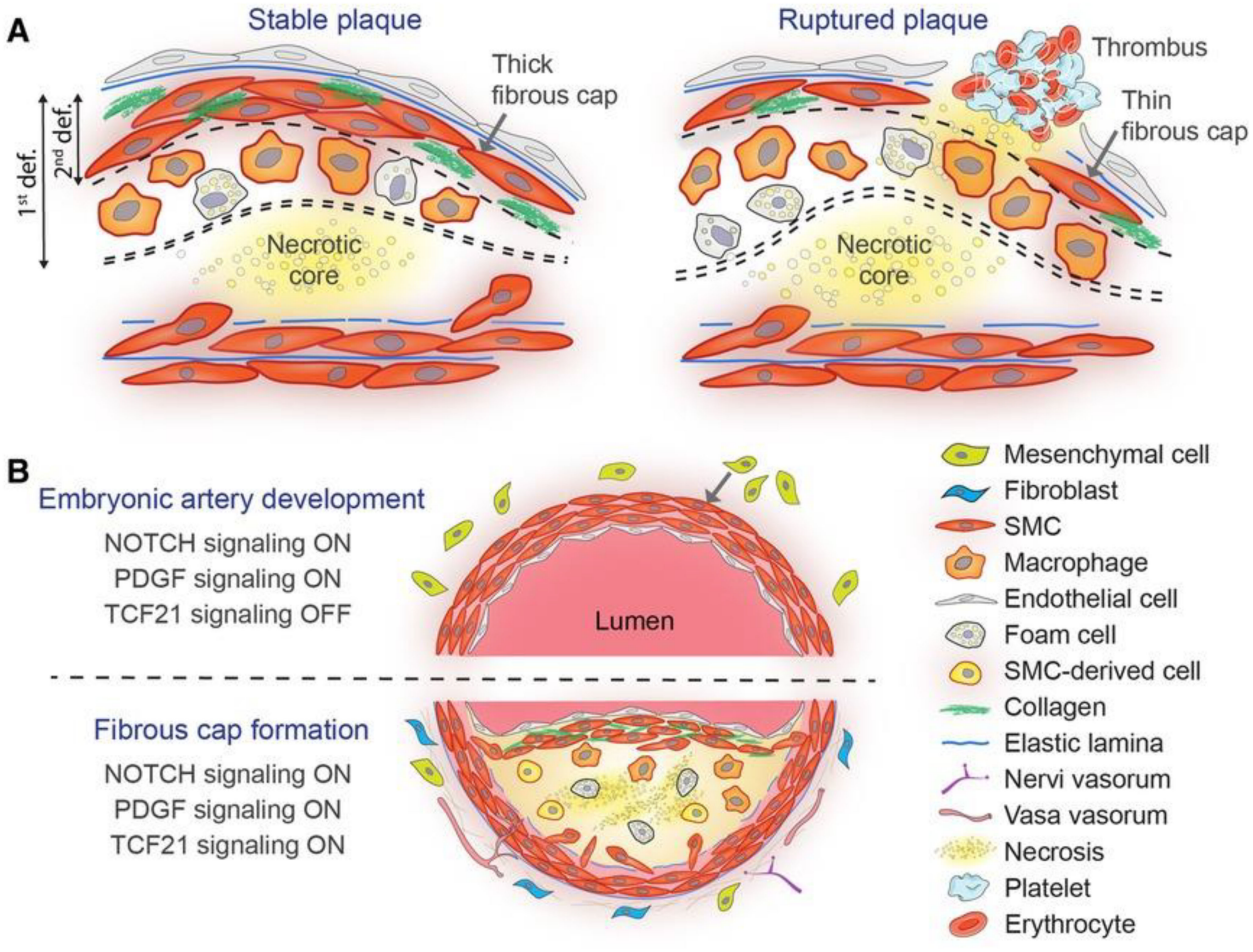
Stable vs. ruptured atherosclerotic plaque **(A)** and similarities between embryonic arterial media development and fibrous cap formation in atherosclerosis **(B)**. **(A)** A simplified scheme depicting the media and neointima of a stable murine lesion with a thick collagen-rich fibrous cap overlying a necrotic core (left). The fibrous cap is highlighted by double or single dashed lines according to the first (i.e., space between the endothelium and the necrotic core) or the second definition (i.e., subendothelial SMCs), respectively. In contrast, lesions with thinner fibrous caps and large necrotic cores are at risk for rupturing and precipitating thrombosis (right). **(B)** NOTCH, PDGF, and TCF21 signaling control SMC recruitment and differentiation during mouse embryonic artery development (top) and fibrous cap formation in atherosclerosis (bottom). Mesenchymal precursor differentiation (either from the neural crest, mesoderm, or the epicardium) towards the SMC fate and away from other lineages (i.e., osteochondrocytes and fibroblasts) requires active PDGF and NOTCH signaling (ON) and silencing of TCF21 (OFF). Fibrous cap SMCs are primarily originated by migration and clonal expansion of medial SMCs and blockade of NOTCH, PDGF, or TCF21 signaling is sufficient to impair cap formation (57).

The spatial organization of this section has been established through depiction of a cross-section of an atherosclerotic plaque in **Figure 2** and includes a necrotic core, a macrophage rich shoulder region and a fibrous cap (57). The plaque architecture shown in **Figure 2** directly maps to ferroptosis-vulnerable compartments. The fibrous cap harbors vascular smooth muscle cells (VSMCs); ferroptosis in VSMCs weakens cap integrity by promoting phenotypic dedifferentiation, reducing collagen synthesis and disrupting extracellular matrix maintenance, as evidenced by selective VSMC ferroptosis inhibition studies that significantly ameliorate atherosclerotic lesions (58). The macrophage-rich shoulder region exhibits high inflammatory activity, abundant oxidized lipoproteins, and dysregulated iron metabolism, creating a niche where labile iron pool expansion and defective efferocytosis converge to lower ferroptosis thresholds and drive necrotic core expansion. The necrotic core provides a catalytically permissive environment with accumulated redox-active iron and phospholipid hydroperoxides that sustain ferroptotic propagation; key mechanisms include ferritinophagy-mediated iron release, NLPR3 inflammasome activation, and impaired clearance of ferroptotic debris (59). Figure highlights NOTCH, PDGF, and TCF21 signaling that regulate SMC differentiation during fibrous cap formation. Notably, ferroptosis has been shown to inhibit NOTCH signaling and influence cell proliferation/differentiation (60). Collectively, each plaque compartment harbors distinct biochemical conditions—catalytic iron availability, peroxidizable phospholipid burden, and antioxidant defense capacity—that determine ferroptosis susceptibility and shape the transition from stable to vulnerable plaque phenotypes.

### Endothelial interface: disturbed flow, oxidative phospholipids, and barrier fragility

Early atherosclerosis develops at arterial sites of disturbed blood flow where the endothelial cells acquire a pro-inflammatory and pro-adhesive phenotype; these areas have oscillating shear stresses that can disrupt normal redox signaling (the balance between oxidative and reductive processes), decrease nitric oxide bioavailability, affect mitochondrial dynamics, and alter metabolic processes that favor ferroptosis (61–63). The endothelial cell’s ability to undergo ferroptosis is highly dependent on the cell’s antioxidant reserves, cystine availability, and the rate at which phospholipid peroxides are generated at membranes. In the context of the initial stages of atherosclerosis, ferroptosis will likely play a role similar to that of an amplifying factor rather than as a primary necrotic core generator. Oxidative stress at the plasma membrane and in organelle membranes can lead to loss of adhesion molecule expression between endothelial cells (i.e., disruption of tight junctions), allow for the migration of leukocytes across the endothelial layer, and promote a pro-thrombotic signal from the endothelium. In addition, the presence of oxidized lipoproteins at the lumen side of the endothelial layer can increase the substrate for peroxidation reactions.

However, iron levels in early-stage atherosclerotic lesions are typically lower than those found in more advanced plaque stages; therefore, endothelial ferroptosis in early stages of atherosclerosis could occur rapidly unless the endothelial cells’ iron homeostasis is disrupted or iron is supplied to the endothelial cells through a variety of mechanisms including microhemorrhage or via iron-rich lipoproteins and heme-related pathways (48). From a mechanistic perspective, early-stage atherosclerosis lesions can be viewed as “detoxification-limited.” That is, if the inflammatory state induced during early-stage atherosclerosis suppresses system Xc- flux, or if the endothelial cells have decreased GPx4 activity relative to the level of lipid peroxides produced, then lipid hydroperoxides can accumulate even in the absence of high levels of iron. Therefore, future studies that investigate the development of early-stage atherosclerosis should consider using ferroptosis-selective inhibitors to assess their ability to restore endothelial barrier function and to prevent the migration of leukocytes into the subendothelial space in response to disturbed blood flow conditions.

### Fatty streak and macrophage foam cell niches: lipid substrate saturation with rising redox complexity

As lesions continue to evolve, monocytes migrate into the intimal layer of the artery wall and undergo differentiation into macrophages; these macrophages take up the modified lipoprotein and then transform into foam cells (64–66). The transformation of the macrophage into a foam cell results in an accumulation of lipid within the cell and alterations in the cell membrane, such as an enrichment of polyunsaturated phospholipids which are involved in ferroptotic execution. There is an interplay between foam cell biology and iron metabolism, since macrophages are responsible for the regulation of iron uptake, storage, export, and recycling, and once activated by inflammation the balance of iron can be shifted toward iron retention and labile pool expansion.

The microenvironment in fatty streak and intermediate lesions contains high levels of oxidized lipids and there is significant inflammatory activity occurring within this environment, both of which enhance the formation of lipid hydroperoxides (67). Therefore, ferroptosis in macrophage-rich areas of fatty streak and intermediate lesions could contribute to the generation of early necrotic debris and amplify the inflammatory response through the release of oxidized phospholipid mediators. It is important to note that macrophage death during the fatty streak and intermediate stages of atherosclerosis is only detrimental when there is ineffective efferocytosis. Effective clearance of dead macrophages converts the macrophage death into a tolerogenic resolution signal. Defective clearance of dead macrophages converts the macrophage death into the initiation of a necrotic core (68, 69). Ferroptosis may be particularly damaging under conditions of defective efferocytosis due to the potential for the iron and oxidized lipid content of dying foam cells to propagate oxidative stress to adjacent cells.

### Advanced plaque core: hypoxia, nutrient constraint, and execution permissiveness

Advanced plaque formation occurs when oxygen diffusion becomes limited due to increased intimal thickness and enhanced metabolic activity of macrophages. The rate of progression of intimal thickness greatly exceeds the rate at which oxygen diffuses into the intima. Therefore, oxygen levels become limiting in many areas of advanced plaques (70). Both inflammation and mitochondrial dysfunction further limit the ability of macrophages within these areas to maintain redox homeostasis. Furthermore, hypoxia and nutrient deficiencies can down-regulate the very metabolic pathways that are protective against ferroptosis. Decreased availability of cystine and decreased capacity to synthesize glutathione as well as alterations in NADPH production and reduced capacity to detoxify phospholipid peroxides can lead to a condition where the basal lipid hydroperoxide load in the plaque core becomes increasingly difficult to buffer.

Concurrently, advanced lesions have accumulated large quantities of oxidized phospholipids, and frequently, iron has deposited in areas of the lesion that correlate spatially with macrophage activity, cell debris and hemorrhage (71). Under such conditions, it is reasonable to suggest that ferroptosis may contribute to the expansion of the necrotic core. The necrotic core is not simply an accumulation of dead cells; it represents a metabolically active compartment that contains large amounts of oxidized lipids, free cholesterol crystals and catalytically active metal ions (72). These components of the necrotic core create an environment that supports continued lipid peroxidative chemistry through positive feedback mechanisms. Under this hypothesis, ferroptosis would function as both an initiator of new necrotic tissue and as an amplifier of the pro-oxidant and pro-inflammatory environment present in the necrotic core.

A major concern for establishing high-confidence claims regarding ferroptosis in advanced lesions will be spatial localization. Specifically, markers of lipid peroxidation and iron enrichment should be localized to the same regions of the plaque core as markers of cell death and dysregulation of ferroptosis regulatory nodes. Studies using spatial omics technologies, laser capture microdissection and/or regional staining and lipidomics approaches will be better positioned to establish that ferroptosis occurs within the plaque core versus representing a systemic oxidative stress response.

### Fibrous cap and shoulder regions: smooth muscle resilience versus vulnerability

The degree to which plaque stability is influenced by the strength of the fibrous cap is dependent upon the ability of vascular smooth muscle cells (VSMC) to synthesize an extracellular matrix (ECM) and organize the ECM in order to maintain the cap’s integrity (73). Vascular smooth muscle cells at the shoulder region of the plaque typically have higher numbers of macrophages and inflammatory mediators than at other regions, which creates both biological and mechanical hotspots for rupture (74). Cap and shoulder regions are subjected to differing pressures that are related to ferroptosis. Vascular smooth muscle cells need to have sufficient antioxidant defenses to survive the conditions of chronic inflammation and lipid stress. However, these cells are exposed to oxidized lipids and cytokines that inhibit the activity of detoxification systems and disrupt the functioning of mitochondria. Although pharmacological inhibition of ferroptosis often preserves fibrous cap integrity, few studies have concurrently satisfied all three validation criteria—particularly criterion 1 (target engagement of GPX4, FSP1, or related regulators)—when interpreting VSMC loss as ferroptotic. Ferroptosis in vascular smooth muscle cells will weaken the scaffold of the cap and increase the likelihood of rupture.

Staging is important in this case because vascular smooth muscle cells may be somewhat protected from ferroptosis in early stages of lesion development; however, as inflammatory mediators, oxidative lipid stress, and local iron availability converge, VSMCs will become increasingly susceptible to ferroptosis. Therefore, there exists a specific niche specificity model where ferroptosis will have the greatest clinical impact when it involves VSMC populations in the fibrous cap and in the transitional areas near macrophage rich shoulders. Thus, demonstrating this hypothesis will require more than just showing that ferroptosis selectively inhibits the death of vascular smooth muscle cells. Instead, it will also be necessary to show that ferroptosis selectively inhibited prevents thinning of the cap, reduces collagen content, or preserves biomechanical surrogates for cap stability in plaque models.

### Intraplaque hemorrhage and erythrocyte derived iron: a catalytic accelerator niche

The presence of intraplaque hemorrhage brings erythrocytes and hemoglobin to the site of the plaque, and consequently, delivers heme iron, thus creating an environment conducive to oxidative stress (75). The potential for direct catalysis of lipid peroxidation exists both from hemoglobin/heme and via the increase in labile iron due to heme’s role as a substrate for pathways of heme degradation. Therefore, this is a region that is highly susceptible to ferroptosis due to its potential to provide the iron-catalyst portion of the ferroptosis triad in a localized and sudden fashion (76, 77). If hemorrhage occurs at a time when the local lipid pool has been oxidized and the ability to detoxify is impaired, the threshold for initiating ferroptosis may be rapidly crossed, leading to increased cell death in the surrounding macrophages and smooth muscle cells.

### Metabolic comorbidities as systemic modifiers of plaque ferroptosis thresholds

Atherosclerotic plaques are influenced by various systemic metabolic states which can create an environment in the plaque microenvironment that is conducive to ferroptosis (78). Insulin resistance and diabetes lead to increased oxidative stress, changes in lipid content, and changes in immune cells’ metabolisms. Chronic kidney disease creates disturbances in iron homeostasis, chronic inflammation, and chronic oxidative stress. Exposure to cigarette smoke leads to an accumulation of oxidized lipids and impairs endothelial cells’ ability to defend against oxidative stress. Therefore, these conditions can be thought of as increasing the amount of basal lipid peroxides generated in addition to decreasing the amount of detoxifying mechanisms available, therefore, reducing the ferroptotic activation threshold in plaque cell types. Mechanistically, this suggests that ferroptosis will not be similarly relevant to all patients suffering from atherosclerosis; rather it may be present at higher levels in those with both iron-related and oxidative stress-related metabolic disturbances.

### Gut microbiota as a remote regulator of plaque ferroptosis thresholds

The gut microbiota has emerged as a non-traditional driver of atherogenesis, with dysbiosis linked to increased intestinal permeability and systemic inflammation (79, 80). Two major classes of microbiota-derived metabolites—trimethylamine N-oxide (TMAO) and short-chain fatty acids (SCFAs)—modulate ferroptosis susceptibility in vascular cells through distinct mechanisms that converge on iron handling, lipid peroxidation, and antioxidant defense pathways.

TMAO, generated from dietary choline and carnitine by gut bacteria with subsequent hepatic oxidation, is an established pro-atherogenic metabolite. Recent evidence directly links TMAO to ferroptosis: TMAO enhances endothelial oxidative stress and promotes foam cell formation through pathways involving inflammasome activation and altered macrophage polarization, processes that share mechanistic overlap with ferroptosis execution (81). In contrast, SCFAs—particularly butyrate—exert protective effects against ferroptosis. Butyrate modulates ferroptosis sensitivity through the FFAR2-mTOR signaling axis, with mechanistic evidence showing that butyrate can both inhibit and, under certain conditions, sensitize cells to ferroptosis depending on the cellular context and concentration (82). Notably, gut-derived SCFAs also promote intestinal barrier integrity, reduce systemic inflammation, and lower oxidative stress burden—all of which contribute to raising ferroptosis thresholds in the arterial wall. Dysbiosis and increased intestinal permeability further influence ferroptosis by enabling translocation of bacterial products that drive systemic inflammation and oxidative stress, while also altering host iron bioavailability. The gut-vascular axis theory posits that restoration of intestinal homeostasis through SCFA supplementation or postbiotic interventions can simultaneously improve gut barrier function and reduce vascular oxidative damage (83).

These insights open new therapeutic possibilities, including dietary fiber/butyrate supplementation, TMAO-lowering strategies (probiotics, FMO3 inhibition), and modulation of gut barrier integrity, as adjuncts to conventional anti-atherosclerotic therapies. Future studies should investigate whether plasma TMAO/SCFA ratios predict plaque ferroptosis activity and whether microbiota-targeted interventions reduce ferroptosis-specific endpoints in human plaques.

## Cell type specific ferroptosis mechanisms in lesions

Ferroptosis mechanisms and consequences vary by cell type, depending on their metabolic functions and local plaque environment. Endothelial cells respond to flow and inflammation; macrophages handle lipids and iron; vascular smooth muscle cells maintain matrix integrity. Each cell type has distinct baseline levels of phospholipids, iron metabolism pathways, antioxidant defenses, and immunomodulatory signaling pathways, which influence which ferroptosis regulatory checkpoints are limiting and what forms of lesions are most susceptible to ferroptotic damage. **Table 2** summarizes ferroptosis determinants, preferred assays, and lesion level outcomes by vascular compartment.

**Table 2:** Cell type specific ferroptosis determinants and lesion phenotypes in atherosclerosis.

Cell type	Dominant ferroptosis drivers in plaque context	Most informative pathway nodes	High specificity readouts	Plaque phenotypes linked to ferroptotic injury	Key confounders and how to control
Endothelial cells (84)	Disturbed flow redox stress, oxidized lipoproteins, inflammatory cytokines, limited cystine availability in stressed niches	system Xc minus, GPX4, NRF2 programs, membrane PUFA remodeling nodes	phospholipid hydroperoxide signatures, rescue by radical trapping antioxidants, GPX4 axis perturbation	barrier dysfunction, leukocyte adhesion and transmigration, pro thrombotic signaling, microvascular leakage into plaque	apoptosis overlap, cytokine driven ROS. Require ferroptosis selective rescue and iron dependence testing
Macrophages and foam cells (85)	oxLDL driven lipid loading, inflammatory activation, iron retention states, heme processing in hemorrhage niches	iron uptake and export, ferritin buffering and mobilization, GPX4 axis, PUFA phospholipid enrichment nodes	labile iron mapping, oxidized phospholipid species, genetic perturbation of ferroptosis gatekeepers	necrotic core seeding and expansion, defective efferocytosis cycles, inflammatory amplification	pyroptosis and necroptosis overlap. Require mode of death discrimination and pathway selective inhibition
Vascular smooth muscle cells (86)	cytokine and oxylipid stress, mitochondrial and redox fragility, local iron exposure near hemorrhage regions, reduced antioxidant reserve in advanced lesions	GPX4, system Xc minus, FSP1 CoQ, PUFA incorporation programs	membrane targeted lipid peroxidation readouts, rescue specificity, preservation of contractile markers	cap thinning, reduced collagen synthesis, plaque mechanical vulnerability	phenotypic switching and senescence. Require linkage to cap metrics and matrix output
Platelets and thromboinflammatory units (87)	oxidative lipid exposure, iron and heme in rupture zones, interaction with NETs and oxidized phospholipids	lipid peroxidation control systems, antioxidant defenses	lipid peroxidation and function assays, inhibitor rescue	thrombogenicity, microthrombi formation at rupture sites	functional readouts can be altered by broad antioxidants. Require careful selectivity controls

### Endothelial cells: ferroptosis as a barrier failure and inflammatory priming module

Endothelial dysfunction is the first measurable step towards the development of atherosclerosis and is a common characteristic of high-risk plaques (88). The endothelium is a unique location where hemodynamics can affect redox signaling and lipid metabolism. Disrupted blood flow patterns will inhibit protective transcriptional pathways while exposing the endothelium to increased amounts of oxidized lipoproteins and inflammatory mediators (89, 90). Exposure to these substances will lead to an increase in phospholipid peroxide burden and within this context, ferroptosis represents a continuum of lipid peroxide stress. Current evidence linking disturbed flow to endothelial ferroptosis predominantly relies on rescue by iron chelators or lipophilic antioxidants (criteria 2 and 3), with comparatively fewer reports directly linking these effects to modulation of canonical regulators such as GPX4 or SLC7A11 (criterion 1). This continuum ranges from sub-lethal lipid peroxide stress that enhances endothelial activation to lethal lipid peroxide stress that disrupts the barrier function of the endothelium.

The key to understanding why endothelial ferroptosis occurs lies with its detoxifying capacity. The ability of endothelial cells to remain viable when exposed to lipid peroxide stress is directly related to the uptake of cysteine through System Xc-1, the availability of glutathione and GPx4 activity. Metabolic reprogramming in response to inflammation or localized nutrient deficiencies can impair this pathway, making it easier for phospholipid hydroperoxides to accumulate. In addition, the remodeling of membranes that increase the content of polyunsaturated fatty acids (PUFAs) can provide additional substrates for peroxidation. When iron becomes available in the vicinity of the endothelial cell, even small quantities, the presence of a large number of phospholipid hydroperoxides, along with the potential to produce PUFAs and/or other peroxidizable substrates, will result in the initiation of free radical chain reactions leading to the occurrence of ferroptotic cell death (29).

In terms of the phenotypic characteristics of the plaque, endothelial ferroptosis would cause a disruption in the barrier function of the endothelium and facilitate the migration of leukocytes into the arterial wall (91). A disrupted barrier function allows for the migration of lipids, cholesterol and monocytes into the arterial wall. In addition, a pro-thrombotic environment would develop due to the loss of nitric oxide mediated anti-platelet signaling and the increased expression of tissue factor on the endothelial surface. Because the injury caused by endothelial ferroptosis can be focal and episodic, the consequences of such an injury may occur as localized areas of barrier disruption rather than widespread loss of endothelial function. Thus, if one is to determine whether endothelial ferroptosis has occurred, one must utilize a combination of functional barrier assays, ferroptosis-selective rescue experiments and localized measures of phospholipid peroxidation and iron localization.

### Macrophages and foam cells: ferroptosis at the intersection of lipid overload, iron recycling, and necrotic core dynamics

Macrophages appear to be pivotal to ferroptosis theories of atherosclerosis due to their ability to incorporate two main factors involved in determining ferroptosis: lipid substrate burden and iron management. Lipid accumulation in foam cells increases intracellular lipid levels and can alter membrane lipid composition, potentially increasing amounts of peroxidizable phospholipids (85, 92). The same macrophage regulates the acquisition, storage and release of iron, and can vary its iron acquisition/iron loss based on inflammatory stimulus. Therefore, areas rich in macrophages (plaque) could be considered high risk for ferroptosis, especially in areas that have an abundance of oxidized lipids and inflammatory mediators. While many studies report iron dependence and phospholipid hydroperoxide accumulation consistent with criteria 2 and 3, genetic or pharmacological targeting of core regulators such as ACSL4 or GPX4 (criterion 1) has been demonstrated in only a subset of models, limiting definitive causal attribution.

In terms of mechanisms involved in macrophage ferroptosis, it is believed that the amount and regulation of the labile iron pool will play a major role. States of iron retention, the ferritin buffering capacity and processes that result in mobilization of iron from stores are all potential ways to increase catalytic activity (93). Additionally, heme processing in hemorrhage-prone lesions can further increase iron availability within the compartment of the macrophage. If the environment created by oxidative stress through the formation of lipid peroxides (through oxidized lipoproteins and inflammatory oxygenation pathways) is sufficient to push the macrophage towards ferroptotic commitment, then antioxidant defenses would need to be inadequate.

Consequences of macrophage ferroptosis depend on the stage and clearance of the lesion (94). Death events in early lesions that have active efferocytosis can be buffered and likely will not lead to significant necrotic cores. However, in late-stage lesions with defective efferocytosis, death due to ferroptosis may contribute to the enlargement of the necrotic core, release oxidized lipids that further stimulate inflammation in the microenvironment and deposition of iron that promotes further lipid oxidation in adjacent cells. This creates a possible positive feedback loop in which ferroptosis stimulates both the oxidized lipid burden and the catalytic iron environment, thus lowering the ferroptosis threshold in adjacent cells.

## Crosstalk with inflammation, immunity, and immunometabolism

### Inflammatory signaling as an upstream modulator of ferroptosis thresholds

Ferroptosis is determined by a set of three factors (availability of catalytic iron; substrate pools of peroxidizable phospholipids; and detoxification) all of which can be changed by inflammation. Inflammatory cytokines and signaling through pattern recognition receptors can change macrophage function to retain iron in some ways: decreasing iron exported from the cell and increasing mobilization and sequestration of iron within the cell, therefore potentially increasing the size of the labile iron pool in certain cases (95, 96). Similarly, endothelial and smooth muscle cells activated in cytokine-rich environments can alter their iron buffering and import programs. All of these changes occur in areas of plaque shoulders, where there is high levels of inflammatory activation.

Inflammation promotes ferroptosis in two ways: (1) it generates lipid peroxides via immune activation and oxidized lipoproteins (87); (2) it impairs detoxification by reducing cystine uptake, glutathione synthesis, NADPH, and mitochondrial redox buffering, thereby weakening the Xc^-^-GSH-GPX4 axis. Therefore, inflammatory signals do not simply coexist with ferroptosis, but rather they establish the biochemical conditions that will determine if ferroptotic death occurs when plaque cells are stressed.

One additional important consideration is that inflammation can have either pro or anti-ferroptotic effects based upon which transcriptional programs are active at any one time. As part of its response to inflammation, the body can activate oxidative stress resistance mechanisms (such as those centered around the transcription factor NRF2) in addition to inducing inflammatory mechanisms (97). Activation of these oxidative stress resistance mechanisms can lead to enhanced resistance to ferroptotic death in two primary ways: by activating genes involved in buffering and detoxifying iron; and by enhancing cellular resistance to oxidative damage. This creates a competitive interaction between the generation of peroxides via inflammatory processes and iron retention, and antioxidant compensatory mechanisms. Ultimately, the ferroptosis susceptibility of individual plaque cells will depend on the relative activity of the competing inflammatory and antioxidant programs.

### Sex-dimorphic regulation of ferroptosis in atherosclerosis

Atherosclerosis exhibits well-documented sex differences in incidence, progression, and plaque phenotype, with young women being relatively protected compared to age-matched men (98). The sharp increase in cardiovascular risk following menopause implicates estrogen as a key protective factor. Recent evidence suggests that these sex differences may be mechanistically linked to ferroptosis susceptibility in vascular cells. Specifically, estrogen can synergistically inhibit ferroptosis in vascular cells, particularly endothelial cells and macrophages, through multiple pathways: activating the NRF2-driven antioxidant system, regulating mitochondrial homeostasis, and upregulating the iron efflux protein ferroportin-1 (FPN1) (99). In ovariectomized ApoE^-^/^-^ mice, estrogen deficiency accelerates atherosclerosis with increased lipid peroxidation and iron deposition; both estradiol and the ferroptosis inhibitor ferrostatin-1 alleviate atherosclerosis by upregulating xCT and GPX4, with NRF2 inhibition attenuating the protective effect of estradiol against endothelial cell ferroptosis.

From our current understanding of the effect of androgen on atherosclerosis, it is obvious that the view that androgen is harmful is too simple. The unstable nature of findings reported in cellular, animal, and clinical studies best demonstrates this (100). Iron metabolism itself is sexually dimorphic. Premenopausal females have lower body iron stores than males, but postmenopausal iron accumulation occurs in tandem with increased cardiovascular risk. Estrogen can directly suppress hepcidin transcription, further modulating systemic iron availability (101). These sex-specific differences in ferroptosis regulation have direct implications for plaque vulnerability: plaques from men exhibit larger lipid-rich necrotic cores and thinner fibrous caps compared to those from women. Future studies should systematically incorporate sex as a biological variable, and sex-stratified therapeutic strategies targeting ferroptosis—such as NRF2 activators or iron chelation tailored to menopausal status—may offer precision medicine approaches to reduce residual cardiovascular risk (99).

### Ferroptosis derived oxidized lipids as inflammatory effectors

Ferroptosis is defined by phospholipid peroxide formation; oxidized phospholipids are not simply inert by-products of lipid oxidation but can be considered bioactive mediators that enhance endothelial activation and recruit leukocytes, and program macrophages for increased inflammation (102). Oxidized phospholipids and their subsequent reactive lipid aldehyde products within plaques will modify receptor signaling, increase cytokine production, and contribute to additional oxidative modification of the plaque. Therefore, ferroptosis can produce an inflammatory response prior to overt cell lysis by sub-lethally altering membrane microdomains and signaling platforms with lipid peroxide stress (103).

This concept is particularly important in regions of macrophage-rich plaques where oxidized lipids will contribute to an enhanced pro-inflammatory environment and inhibit efferocytosis, contributing to poor clearance of dying cells and an enlarged necrotic core.

### Innate immune sensors and death pathway interactions

Atherosclerosis encompasses many regulated forms of cell death and the interaction between these pathways will ultimately determine how much damage will occur in the plaque. Lipid stress leads to mitochondrial dysfunction, and both lead to the production of pro-inflammatory cytokines via activation of inflammasomes, particularly those belonging to the NLR family (104, 105). Cell death due to pyroptosis and necroptosis can result in cellular membrane rupture and amplify the release of pro-inflammatory cytokines, and therefore, share similar signs with ferroptosis found in oxidative lesions.

Ferroptosis and inflammatory death pathways can communicate with each other at three different levels (106). First, lipid peroxidation due to ferroptosis can generate lipid danger signals and disrupt the integrity of organelles, thus promoting the activation or priming of innate immune responses. Therefore, ferroptotic lipid peroxidation can also serve as an upstream trigger for innate immune activation. Second, inflammatory death pathways can increase oxidative stress and alter the regulation of iron metabolism, thereby lower the threshold for ferroptosis in surrounding cells. Third, a single lesion site may have multiple modes of cell death occurring simultaneously. It is therefore essential to avoid solely attributing cell death and subsequent inflammation to ferroptosis.

## Biomarkers, imaging surrogates, and human translational evidence

Translating ferroptosis as a mechanism to an actionable pathway for atherosclerosis, will require credible evidence of human disease with the development of adequate biomarkers to specifically identify ferroptosis from general oxidative stress. The translation process will be difficult due to the nature of plaques being inherently oxidative. An increased amount of many common oxidative markers have been found in atherosclerosis; however, this does not imply ferroptotic execution. The most informative translational signals for ferroptosis are indicators of the defining biochemical dependencies: accumulation of phospholipid hydroperoxides in membrane lipids, catalytic iron permissiveness, and suppression or remodeling of ferroptosis suppressor systems. Therefore, this section reviews candidate biomarkers and imaging correlates based on specificity, spatial localization and clinical interpretation, and subsequently presents a translational pipeline for validation. Applying the three-domain validation framework, most human plaque studies fulfill only one or two domains—commonly iron accumulation or oxidative lipid signatures—without concurrent demonstration of target engagement at the level of GPX4, SLC7A11, or related regulators.

### Principles for ferroptosis biomarker credibility in atherosclerosis

There are three rules to assess the validity of ferroptosis biomarkers for arterial diseases (107). The first rule is to prioritize specificity for ferroptosis to the extent possible over sensitivity to oxidative stress. Broadly reactive lipid oxidation biomarkers may be acceptable if they can be selectively rescued by ferroptosis-specific mechanisms or indicate pathway checkpoint failure. Second, spatial correlation is important; plaque biology is highly compartmentalized, so it would be more persuasive if a biomarker were found in compartments known to have high levels of ferroptosis (e.g., macrophage-rich shoulders, hemorrhage “niches,” or fibrous cap smooth muscle zones). Third, biomarkers should correlate with specific clinical manifestations of vascular disease including plaque instability, intraplaque hemorrhage, and symptomatic vascular events.

In terms of operationally evaluating human data, the most compelling will be those that support at least two of the following criteria: iron accumulation consistent with catalytic permissive conditions, enrichment of phospholipid peroxidation products by ferroptosis-associated lipid species, and alterations in either abundance or activity of the ferroptosis regulatory molecules (GPX4 axis) or related suppressive pathways. Claims of single biomarkers should be viewed as hypothesis-generating.

### Status of direct human plaque evidence

The causal relationship between iron and atherosclerosis has long been debated. A 2023 review critically examined why patients with hereditary hemochromatosis—characterized by systemic iron overload—do not show increased atherosclerosis incidence, arguing that iron homeostasis within the arterial wall itself is the critical determinant and supporting a causal link between local iron accumulation and disease progression (108). Extending molecular insights to clinical imaging, an analysis of 300 patients using coronary CT angiography found that higher iron levels were associated with the presence of low-attenuation plaque and a greater percentage of vulnerable plaque components, providing direct *in vivo* human evidence linking iron burden to plaque instability (109). The mechanistic link between iron accumulation and ferroptosis in vulnerable plaques is further elaborated through discussions of intraplaque erythrophagocytosis and heme oxygenase-1 overexpression as critical drivers of iron-mediated lipid peroxidation (76). Collectively, these studies provide suggestive evidence that ferroptosis-related pathway alterations correlate with plaque vulnerability in human tissues. However, none have simultaneously quantified phospholipid hydroperoxides, labile iron pools, and functional GPX4 activity in the same plaque compartments—a triad required for definitive ferroptosis attribution. Thus, while correlative human data are increasingly available, definitive proof of execution-phase ferroptosis in patient tissues remains an unmet need.

## Therapeutic targeting, limitations, challenges, future directions

Ferroptosis has been an attractive therapeutic area for atherosclerosis due to its biochemical similarity to how plaques evolve. Atherosclerotic lesions contain a higher concentration of oxidizable phospholipids and oxidized lipoproteins, have pro-inflammatory and low oxygen conditions (microdomains) which reduce antioxidant availability, and in many of these high-risk plaques there is increased redox active iron accumulation via abnormal iron regulation or heme turnover from bleeding. These factors provide a reasonable basis for the potential for oxidative stress (lipid hydroperoxides) to exceed the capacity of cells to detoxify and induce regulated lipid dependent cell death. Translational-ly the main advantage is to inhibit a pathway that may be involved in endothelial injury, macrophage foam cell dysfunction, necrotic core enlargement and smooth muscle depletion in the fibrous cap of the plaque. The main disadvantage is that both iron metabolism and lipid peroxide control are systemic requirements. Therefore, any intervention will need to demonstrate a positive effect at the level of the lesion while minimizing any negative “off-target” effects and demonstrate that it modulates ferroptosis as opposed to having non-specific antioxidant activity.

Based on the document, therapeutic strategies targeting ferroptosis in atherosclerosis can be conceptualized into four main classes, each addressing a different node of the pathway with specific translational considerations. The first strategy aims to reduce iron’s catalytic permissiveness by depleting the labile iron pool within plaques; however, systemic iron chelation is limited by side effects like anemia, shifting focus towards precision therapies that target iron-rich plaque niches (e.g., hemorrhagic areas), with success defined by the reduction of both labile iron and ferroptotic lipids *in situ*. Systemic GPX4 activation carries potential pro-atherogenic risks. In ApoE^-^/^-^ mice, GPX4 overexpression reduced plaque lipid hydroperoxides but did not alter plaque size, necrotic core area, or fibrous cap thickness. This dissociation suggests that simply boosting GPX4 activity is insufficient to stabilize plaques and may inadvertently maintain pro-inflammatory cell states. Furthermore, GPX4 knockdown suppresses M2 (anti-inflammatory) macrophage polarization in cancer models, implying that GPX4 overexpression might favor pro-inflammatory phenotypes (110, 111). Thus, GPX4-targeted therapies for atherosclerosis likely require cell-type-specific delivery, combination with anti-inflammatory agents, and patient stratification based on plaque inflammatory status.

The second class inhibits the propagation of phospholipid peroxides using lipophilic radical-trapping antioxidants. The historical failure of broad antioxidants in cardiovascular trials underscores the necessity for new agents to specifically suppress ferroptosis-associated phospholipid hydroperoxides within plaques, using specific molecular readouts to distinguish their effect from general antioxidant or anti-inflammatory actions. The third approach reinforces endogenous defense systems, such as enhancing the system Xc-/GSH/GPX4 axis or parallel pathways like FSP1/CoQ. A key challenge here is the systemic role of these pathways; convincing evidence for such therapies requires demonstrating a specific reduction in membrane phospholipid hydroperoxides and the preservation of plaque-stabilizing cells like smooth muscle cells. A fourth, indirect strategy involves modulating upstream risk factors, such as through intensive lipid-lowering or anti-inflammatory treatments, which may reduce ferroptosis risk by decreasing oxidizable substrates or inflammatory peroxide production. While promising, establishing that ferroptosis inhibition is a direct causal mechanism in the benefits of these established therapies requires combining them with the measurement of specific biomarkers that reflect the activity of ferroptosis execution and defense checkpoints within the atherosclerotic lesion itself.

It is also important to pay attention to the therapeutic effect of NRF2 on atherosclerosis. NRF2 activation suppresses ferroptosis by upregulating SLC7A11, GPX4 and iron-sequestering genes, raising ferroptotic thresholds. However, NRF2’s effects in atherosclerosis are context-dependent. Global Nrf2 deficiency in hypercholesterolemic mice paradoxically reduces lesion size but promotes plaque instability, including thinner fibrous caps, increased inflammation and spontaneous myocardial infarction (112). Conversely, NRF2-mediated ferroptosis inhibition may preserve vascular smooth muscle cells and cap integrity. Moreover, systemic NRF2 activation can enhance foam cell formation via CD36 upregulation and alter hepatic cholesterol metabolism, potentially accelerating early lesion progression. Thus, while NRF2 activation is a promising ferroptosis-suppressive strategy, cell-type-specific delivery is required to avoid metabolic side effects. Timing also matters: early activation may limit lesion initiation, while late-stage activation could stabilize vulnerable plaques (113).

There are several areas in which current evidence must be improved prior to ferroptosis targeting being considered clinically mature. There is currently a lack of consistent operational definitions among studies, many of which utilize nonspecific lipid peroxidation markers or broad antioxidant responses that do not demonstrate iron dependency or selective rescue. There is also a lack of uniform specificity among pharmacological tools to modulate ferroptosis and there are many ways in which they can affect inflammation and metabolism independently of ferroptosis, thus requiring both genetic and lipidomic validation for strong inference. A central limitation is the infrequent application of all three validation criteria—target engagement, iron dependence, and phospholipid hydroperoxide quantification—within individual studies, resulting in mechanistic claims that are often correlative rather than causative. Additionally, studies are frequently insufficiently characterized with regard to the stage and niche specificity of the lesions, and while ferroptosis is expected to be prevalent in iron- and oxylipid-rich microenvironments, such as hemorrhage-prone regions and macrophage-rich shoulders, it is not expected to be uniformly present throughout all plaques. There is also a lack of complete validation in humans, particularly regarding the demonstration of colocalization of ferroptosis-executor lipids with catalytically active iron and failed checkpoints in plaque-vulnerable regions.

A further critical gap is the absence of strategies for cell-type-selective ferroptosis modulation in plaques. Endothelial cells, macrophages, and vascular smooth muscle cells (VSMCs) have distinct ferroptosis sensitivities and divergent roles in plaque (in)stability. Thus, a “one-size-fits-all” systemic inhibitor may simultaneously protect VSMCs (beneficial) while inadvertently preserving pro-inflammatory macrophage foam cells (deleterious), depending on disease stage. Defining cell-type-specific therapeutic windows—e.g., early intervention to limit endothelial ferroptosis versus late-stage targeting of macrophage ferroptosis to reduce necrotic core expansion—is essential but remains unexplored. Nanoparticle-based delivery offers a path forward: liposomes or polymeric nanoparticles functionalized with antibodies against CD36, SR-A, or VCAM-1 can selectively target foam cells or inflamed endothelium. ROS- or pH-responsive nanocarriers enable triggered release within the oxidative plaque microenvironment. Furthermore, siRNA or CRISPR vectors packaged into cell-type-specific vehicles could achieve genetic ferroptosis checkpoint modulation without systemic toxicity. However, nanoparticle uptake by phagocytes in the liver and spleen remains a major hurdle, necessitating advanced stealth coatings or pre-targeting strategies. Future work should prioritize head-to-head comparisons of cell-type-selective versus systemic ferroptosis inhibitors, using lineage-traceable models to dissect which cell’s ferroptosis drives adverse outcomes at each lesion stage. Finally, atherosclerotic plaques are composed of multiple regulated cell death modalities, and distinguishing between ferroptosis and other forms of programmed cell death (pyroptosis, necroptosis, and apoptosis) is crucial for establishing causal relationships.

Future advances are likely to depend on developing standardized criteria for establishing ferroptosis attribution in vascular research studies, including phospholipid hydroperoxide-level readouts, iron-dependency testing, and selective rescue in combination with genetic perturbation of critical regulatory molecules. Establishing cell-type and stage-resolved causality will require conditional genetic manipulation of endothelial cells, macrophages, and smooth muscle cells, and correlation of plaque composition and stability metrics rather than only cell death markers. Defining the location and extent of ferroptosis activity within human plaque tissue will require multimodal mapping techniques, including spatial transcriptomics, iron localization, and lipidomics, to identify the relationship of ferroptosis activity to plaque instability phenotypes. Development of clinical biomarkers should be tissue-based, with plasma-based markers validated against plaque-based signatures and imaging surrogate markers of hemorrhage or iron enrichment to facilitate patient stratification. Drug design should preferentially focus on delivering therapeutics to the lesion site or selectively modulating specific cell types to maximize the safety margin.

## Conclusion

Atherosclerotic disease processes occur within the wall of arteries that are rich in oxidizable phospholipids, chronic inflammation and redox dysregulation. Therefore, ferroptosis, an iron-dependent regulated cell death process initiated by nonenzymatic phospholipid peroxidation may play a mechanistic role in the progression and instability of lesions. Ferroptotic stress likely affects atherosclerosis through several pathways that are specific to various cell types; for example, ferroptotic stress may cause disruptions in the endothelial barrier function and recruitment of leukocytes, induce macrophage/foam cell death resulting in the growth of the necrotic core as well as amplify oxidized lipids when there is defective efferocytosis, and induce vascular smooth muscle cell loss leading to compromised fibrous cap integrity and increased rupture susceptibility. However, despite this potential, translation of ferroptosis to human atherosclerosis has been limited due to inconsistent definition of functional ferroptosis and widespread use of nonspecific oxidative stress markers (that do not confirm iron dependency, selective rescue and membrane phospholipid hydroperoxide executors) to distinguish ferroptosis from other forms of oxidative damage. A context dependent model is the best fit for current data, where ferroptosis will be most relevant in spatially defined, high risk plaque niches with both catalytic iron availability and oxidized lipid burden and limited antioxidant defense capacity. For future research, it would be best to have standardized criteria for identifying ferroptosis in all cell types, link causality testing of cell targeted interventions to plaque stability end points, and perform multimodal spatial validation of ferroptosis in human plaques using methods such as iron localization, oxidized phospholipid chemistry and checkpoint status. Finally, development of tissue anchored biomarkers and lesion selective therapeutic approaches that reduce the levels of ferroptosis executors with a safe and tolerable systemic toxicity profile is also required.
